# “BUT WE ARE NOT POWERLESS AGAINST THIS PROBLEM”: OLDER PEOPLE, LONELINESS AND THE MEDIA

**DOI:** 10.1093/geroni/igz038.1367

**Published:** 2019-11-08

**Authors:** Mary Pat Sullivan

**Affiliations:** Nipissing University, North Bay, Ontario, Canada

## Abstract

Media campaigns play a critical role in framing public perceptions or ‘public talk’ around social issues. The media’s role in characterizing the loneliness ‘problem’ is, however, an under explored area. This paper presents the language of loneliness and loneliness representations in the media in Canada and England over a 10-year period (2009-2018) and their relationship with key policy initiatives specific to an ageing population. Using qualitative content analysis, the findings illustrate the use of skilled marketing techniques and highly stigmatizing discourse. These media approaches act to: (1) reinforce the threat of an ageing population; (2) endorse responsibilization and governmentality of the body; and (3) promote individual and/or family shame and morally responsible actions by charities and volunteers. We conclude that there is a need for a critical analysis of loneliness from the perspective of social and cultural constructions of ageing, the positioning of older people in society, and neo-liberalist ideology

